# Virulence Determinants, Antimicrobial Resistance, and Biofilm Formation of *Staphylococcus aureus* Recovered from Ready-to-Eat Foods and Food Handlers in University Food Services

**DOI:** 10.3390/foods15132331

**Published:** 2026-07-01

**Authors:** Kamila Soares, Manuela Matos, Joana Paiva, Marlene Santos, Sónia Saraiva, Juan García-Díez, Alexandra Esteves, Cristina Saraiva

**Affiliations:** 1 Department of Veterinary Sciences, University of Trás-os-Montes and Alto Douro (UTAD), 5000-801 Vila Real, Portugal; 2 Animal and Veterinary Research Centre (CECAV), University of Trás-os-Montes and Alto Douro (UTAD), 5000-801 Vila Real, Portugal; 3 Associate Laboratory of Animal and Veterinary Sciences (AL4AnimalS), 5000-801 Vila Real, Portugal; 4 Department of Genetics and Biotechnology, University of Trás-os-Montes and Alto Douro (UTAD), 5000-801 Vila Real, Portugal; 5 Centre for the Research and Technology of Agro-Environmental and Biological Sciences (CITAB), University of Trás-os-Montes and Alto Douro (UTAD), 5000-801 Vila Real, Portugal; 6 Institute for Innovation, Capacity Building and Sustainability of Agri-food Production (Inov4Agro), 5000-801 Vila Real, Portugal

**Keywords:** *Staphylococcus aureus*, food safety, ready-to-eat foods, food handlers, antimicrobial resistance, virulence genes, biofilm formation

## Abstract

*Staphylococcus aureus* is a major food safety concern because of its ability to produce heat-stable enterotoxins, develop antimicrobial resistance, and express virulence factors associated with persistence and pathogenicity. The present study characterised *S. aureus* isolates recovered from ready-to-eat (RTE) foods and food handlers’ hands in university food service establishment in northern Portugal, focusing on virulence-associated genes, antimicrobial resistance profiles, and biofilm production. A total of 261 samples were analysed, including 156 RTE food samples and 105 hand swabs. Twenty-nine coagulase-positive staphylococci isolates were recovered and confirmed as *S. aureus* by detection of the *nuc* gene, corresponding to an overall prevalence of 11.11% (29/261). Of these, 20 isolates were obtained from food handlers’ hands and 9 from RTE foods. The *hla* and *sei* genes were detected in all isolates, while *seg* and *tst* were detected in 93.10%; *sed* was not detected. Biofilm-forming capacity was identified in 44.83% of isolates, with most strains exhibiting weak to moderate biofilm production. Resistance to at least one antimicrobial agent was observed in 31.0% of isolates, and presumptive methicillin-resistant *Staphylococcus aureus* represented 13.79%, all classified as multidrug-resistant. These findings support the occurrence of handling-related contamination and reinforce the need for strict hygiene practices, temperature control, and continuous monitoring in institutional food service environments.

## 1. Introduction

*Staphylococcus aureus* is a Gram-positive bacterium frequently arranged in grape-like clusters and capable of growing under both aerobic and anaerobic conditions, with optimal growth occurring at approximately 37 °C and neutral pH [1].

*S. aureus* is a common pathogen associated with health risks because of its capacity to produce enterotoxins and acquire antimicrobial resistance (AMR) [2]. These virulence determinants are generally divided into surface-associated components, which facilitate adhesion and immune evasion, and extracellular factors, including toxins and enzymes that contribute to host tissue damage and disease progression. Staphylococcal toxins are commonly classified into three categories: (i) toxins that damage the cytoplasmic membrane, either receptor-mediated or independent; (ii) toxins that interfere with membrane receptor function; and (iii) secreted enzymes with degradative activity [3]. Among membrane-damaging toxins, alpha-hemolysin, encoded by the *hla* gene, is one of the most investigated and is present in a high proportion of *S. aureus* strains. This toxin induces lysis of erythrocytes and leukocytes and has been associated with severe infections, including pneumonia, sepsis, and skin infections [4,5]. However, the detection of the *hla* gene does not necessarily indicate active toxin production, as its expression is tightly regulated by environmental conditions and through regulatory networks including the accessory gene regulator (*agr*) and *Staphylococcus aureus* exoprotein expression regulator (*SaeRS*) [5,6].

Staphylococcal enterotoxins (SEs) represent a most important concern in food safety because of their emetic proprieties and their role in staphylococcal food poisoning (SFP) [7,8]. These toxins are characterized by their high stability, remaining active after exposure to heat, proteolytic enzymes, and a wide range of pH conditions [9]. They belong to the superantigen family owing to their capacity to trigger extensive activation of T lymphocytes, leading to cytokine release and, in some cases, contributing to toxic shock syndrome (TSS), which may result in severe systemic disease [10,11]. The classical staphylococcal enterotoxins (*sea*, *seb*, *sec*, *sed*, and *see*) are well-established contributors of SFP, whereas the role of newly described enterotoxins and enterotoxin-like proteins (SEls) remains less clearly defined [12,13]. The enterotoxin gene cluster (*egc*), which includes genes such as *seg* and *sei*, is frequently detected in *S. aureus* isolates and has been associated with increased pathogenic potential [14,15].

In addition to toxin production, biofilm formation by *S. aureus* contributes to important role its persistence and dissemination in food service environments. Biofilms consist of structured microbial communities associated with biotic or abiotic surfaces and surrounded by an extracellular matrix produced by the cells. The extracellular matrix promotes bacterial survival under adverse conditions and may confer markedly increased tolerance to disinfectants and antimicrobial agents compared with planktonic cells [16,17]. In food service environments, biofilm formation may contribute to persistence on food-contact surfaces and may contribute to recurrent contamination events [18].

The increased occurrence and spread of antimicrobial-resistant *S. aureus*, especially methicillin-resistant strains (MRSA), further increases its relevance to public health. Antimicrobial resistance is mediated by a range of mechanisms, including alterations of antimicrobial target sites, enzymatic drug inactivation, active efflux systems, and gene exchange mechanisms involving mobile genetic elements, including plasmids [19,20]. The occurrence of multidrug-resistant (MDR) strains within the food chain is considered a concern, due to their potential to spread resistance genes across humans, animals, and environmental reservoirs, contributing to the spread of antimicrobial resistance in a One Health context [21].

*S. aureus* is commonly found as a commensal organism on human skin and mucous membranes, with approximately 20 to 30% of individuals being persistent carriers [22]. Food handlers therefore represent a relevant source of contamination in ready-to-eat (RTE) foods, where no further heat treatment is applied prior to consumption. Contamination may occur through direct contact, inadequate hand hygiene, or improper food handling practices, increasing the risk of foodborne disease transmission [23,24].

Foodborne diseases affect approximately 600 million people annually [25]. In the European Union, *S. aureus* toxins are among the leading causes of foodborne outbreaks associated with catering settings, including canteens and workplace food services [26]. These outbreaks are frequently linked to inadequate temperature control, cross-contamination, and the involvement of colonized food handlers.

Despite the recognized importance of *S. aureus* in food safety, integrated studies assessing antimicrobial resistance, virulence determinants, and biofilm-forming capacity in institutional food service settings remain limited. Therefore, this study aimed to characterize *S. aureus* isolates recovered from RTE foods and food handlers’ hands in university food service establishments in Northern Portugal, focusing on antimicrobial resistance profiles, virulence-associated genes (*nuc*, *hla*, *tst*, *sed*, *seg*, and *sei*), and biofilm-forming capacity.

## 2. Materials and Methods

### 2.1. Characterization of the Establishments and Sampling

Sampling was conducted on RTE foods and food handlers’ hands at two university locations in northern Portugal. A total of 156 RTE food samples were collected, including 96 samples from university 1 and 60 samples from university 2. Additionally, 105 food handlers were sampled, corresponding to approximately 90% of the workforce, with 57 individuals from university 1 and 48 from university 2, each providing one hand swab sample (*n* = 105).

For food sample collection, 250 g of each RTE food item was aseptically placed in sterile plastic bags and transported to the laboratory within one hour. Samples were stored at 3 °C and processed within 24 h, in accordance with ISO 6887-1:2017 [27].

### 2.2. Microbiological Analysis

All microbiological procedures were performed under aseptic conditions.

For hand swab samples, a sterile swab moistened with 10 mL of peptone solution (Himedia, Mumbai, India) was rubbed over the palm and fingers of the dominant hand for approximately 20 s, in accordance with ISO 18593:2018 [28]. Each swab was vortexed for 60 s (Fisherbrand, Fisherbrand, PA, USA), and serial decimal dilutions were prepared in peptone salt solution. For enumeration, 0.1 mL aliquots of appropriate dilutions were spread onto Baird–Parker agar (BP, Biolab, Budapest, Hungary) supplemented with egg yolk tellurite emulsion (VWR, Leuven, Belgium) and incubated at 37 °C for 48 h, following ISO 6888-1:2021 [29]. For detection, 1 mL of the initial swab suspension was transferred into 9 mL of Giolitti–Cantoni enrichment broth (Himedia, Mumbai, India) supplemented with potassium tellurite solution. After incubation at 37 °C for 24–48 h, 0.1 mL of the enriched suspension was plated onto Baird–Parker agar supplemented with egg yolk tellurite emulsion and incubated aerobically at 37 °C for 24–48 h.

For food samples, 10 g of each item was homogenised with 90 mL of peptone solution (Himedia, Mumbai, India) using a stomacher (Lab Blender, Seward, Worthing, West Sussex, UK) for 60 s, in accordance with ISO 6887-1:2017 [27]. Serial decimal dilutions were prepared in peptone salt solution and analysed using the same enumeration and detection procedures described for hand swab samples adapted. To confirm the presence of coagulase-positive staphylococci (CoPS), the tube coagulase test was performed according to ISO 6888-1:2021 [29]. Suspected colonies were inoculated into 1 mL of Brain Heart Infusion (BHI) broth and incubated at 37 °C for 24 h. Subsequently, 0.1 mL of culture was transferred into sterile tubes containing 0.3 mL of rabbit plasma with fibrinogen (Biolife, Monza, Italy). Tubes were gently mixed and incubated at 37 °C, with clot formation assessed after 4 h and 24 h. Samples showing a firm and stable clot were considered positive. *Staphylococcus aureus* ATCC^®^ 29213 and *Staphylococcus epidermidis* ATCC^®^ 12228 were used as positive and negative controls, respectively.

All confirmed positive isolates were preserved at −80 °C.

#### Storage and Regeneration of Bacterial Cultures

A total of 29 CoPS isolates from foods and food handlers were stored at −80 °C in Brain Heart Infusion (BHI) broth (Himedia, Mumbai, India) supplemented with 15% (*v*/*v*) glycerol (Liofilchem, Roseto degli Abruzzi, Italy) for subsequent analysis.

For regeneration, frozen cultures were thawed and inoculated into BHI broth, followed by incubation at 37 °C for 24 h. Once visible turbidity was observed, isolates were subcultured onto Aureus ChromoSelect agar (Millipore, Mumbai, India) supplemented with egg yolk tellurite emulsion (VWR, Leuven, Belgium) and incubated at 37 °C for 24 h to obtain pure colonies.

### 2.3. Genetic Analysis and DNA Extraction

Following regeneration and isolation, CoPS isolates were used for DNA extraction. Genomic DNA was isolated using a commercial kit (GF-1 Bacterial DNA Extraction Kit, Vivantis, Subang Jaya, Selangor, Malaysia) following the manufacturer’s recommendations and stored at −20 °C for subsequent analyses.

DNA integrity was evaluated by 1% agarose gel electrophoresis. DNA concentration and purity were determined using a spectrophotometer (BioTek PowerWave XS2, BioTek Instruments, Winooski, VT, USA) coupled with Gen5 software (version 3.05). Absorbance was recorded at 260 nm and 280 nm, and DNA concentration and purity were calculated based on A260 and the A260/A280 ratio.

#### Detection of Enterotoxin and Virulence Factors

The thermonuclease gene (*nuc*) was selected as a target for the *S. aureus* identification. PCR assays were performed using specific primers for *nuc*, *hla*, and *tst* genes in separate reactions. The detection of SE genes was accessed by multiplex PCR using specific primers for *sed*, *seg*, and *sei*.

PCR reactions were carried out in an Applied Biosystems 2720 Thermal Cycler (Applied Biosystems, Foster City, CA, USA). PCR amplifications were performed in a final volume of 20 μL containing 10.0 μL of Taq Master Mix MyGo HS, 0.5 μL of each primer (10 μM), and 6.0 μL of DNA (10 ng/μL). Free nuclease water was used as a negative control, and DNA from *S. aureus* ATCC^®^ 29213 was used as a positive control for *nuc* gene amplification. DNA from *nuc*-positive *S. aureus* isolates was subsequently used as positive control material in the remaining PCR assays. The PCR assays were performed using previously published and validated primer sets and amplification conditions [30,31,32,33]. Amplification products were evaluated according to their expected amplicon sizes by agarose gel electrophoresis. PCR cycling conditions consisted of an initial denaturation at 95 °C for 5 min, followed by 35 cycles of three-step amplification: 95 °C for 15 s, annealing at the appropriate temperature (Table 1) for 20 s, and 72 °C for 20 s, with a final extension at 72 °C for 5 min.

Different agarose gel concentrations were used depending on the target gene: 1% for *nuc*, 1.2% for *hla* and *tst*, and 1.5% for *sed*, *seg*, and *sei*. Primer sequences, amplicon sizes, and annealing temperatures are presented in Table 1.

### 2.4. Biofilm Formation Assay

The biofilm formation assay was performed with modifications proposed by Silva et al. [17]. Two colonies from fresh bacterial cultures were transferred to 3 mL of Brain Heart Infusion (BHI, Himedia, India) broth and incubated at 37 °C for 24 ± 2 h. Following incubation, the bacterial suspension was adjusted to a 0.5 McFarland turbidity standard. Aliquots of 200 µL were then inoculated into individual wells of a 96-well flat-bottom microtiter plate. *Staphylococcus aureus* ATCC^®^ 29213 served as the positive control, while uninoculated sterile medium was used as the negative control. Plates were incubated under static conditions at 37 °C for 24 h, and all experiments were performed in technical replicates.

#### 2.4.1. Assessment of Biofilm Biomass

Biofilm biomass was assessed using the Crystal Violet (CV) staining assay, following the procedure described by Peeters et al. [34], with minor modifications. After incubation, the plates were washed twice with 200 µL of distilled water to remove non-adherent cells and allowed to dry at room temperature for approximately 1 h. Subsequently, 100 µL of methanol (VWR International, Carnaxide, Portugal) was added to each well and incubated for 15 min to fix the biofilm. After removing the methanol, the plates were dried at room temperature for 10 min. Then, 100 µL of 1% (*v*/*v*) CV (Liofilchem, Roseto degli Abruzzi, Italy) was added to each well for 10 min.

The excess dye was removed by washing the plates twice with distilled water. Finally, 100 µL of 33% (*v*/*v*) acetic acid (Chem-Lab NV, Zedelgem, Belgium) was added to solubilize the CV, and the absorbance was measured at 570 nm using a BioTek PowerWave XS2 microplate reader (BioTek Instruments, Winooski, VT, USA).

#### 2.4.2. Classification of Biofilm Production

Isolates were assigned to one of four categories according to their biofilm-forming capacity—non-producer, weak, moderate, or strong producer—based on optical density measurements at 570 nm (OD570). The cut-off value (ODc) corresponded to the mean optical density negative controls plus three standard deviations. Isolates were classified as non-producers (OD ≤ ODc), weak (ODc < OD ≤ 2 × ODc), moderate (2 × ODc < OD ≤ 4 × ODc), and strong (OD > 4 × ODc) biofilm producers.

To facilitate comparisons among independent experiments, OD values were normalised using *Staphylococcus aureus* ATCC^®^ 29213 as the reference strain, which was included in every assay.

### 2.5. Antimicrobial Susceptibility Testing

Pure cultures of *S. aureus* were evaluated for antimicrobial susceptibility using the Kirby–Bauer disk diffusion assay. A panel of 14 antimicrobial agents (Oxoid, Thermo Fisher Scientific, Basingstoke, UK) was tested.

For each isolate, five to six colonies from overnight growth were suspended in 0.9% NaCl solution and adjusted to a turbidity equivalent to a 0.5 McFarland standard. The resulting inoculum was evenly spread onto Mueller–Hinton agar plates (Oxoid, Hampshire, UK). Antibiotic discs containing penicillin G (P, 10 U), cefoxitin (FOX, 30 µg), chloramphenicol (C, 30 µg), ciprofloxacin (CIP, 5 µg), clindamycin (DA, 2 µg), erythromycin (E, 15 µg), gentamicin (CN, 10 µg), imipenem (IPM, 10 µg), linezolid (LNZ, 10 µg), mupirocin (MUP, 200 µg), oxacillin (OX, 1 U), rifampicin (RD, 30 µg), tetracycline (TE, 30 µg), and trimethoprim/sulfamethoxazole (SXT, 1.25/23.75 µg) were applied to the agar surface.

Following incubation at 35 ± 1 °C for 18 ± 2 h, inhibition zone diameters were measured in millimetres and interpreted according to EUCAST criteria [35] for penicillin G (benzylpenicillin), oxacillin, cefoxitin, gentamicin, trimethoprim/sulfamethoxazole, mupirocin, rifampicin, linezolid, tetracycline, erythromycin, clindamycin, and ciprofloxacin. For imipenem and chloramphenicol, interpretation was based on Clinical and Laboratory Standards Institute (CLSI) guidelines [36], as EUCAST disk diffusion breakpoints for *S. aureus* were not available. *Staphylococcus aureus* ATCC^®^ 29213 was used as a quality control strain.

### 2.6. Statistical Analysis

Data analysis was carried out using IBM SPSS Statistics, version 31.0 (IBM Corp., Armonk, NY, USA). Differences in *S. aureus* prevalence and antimicrobial resistance rates between isolates recovered from foods and food handler’s hands were examined using Fisher’s exact test. Odds ratios (OR) with corresponding 95% confidence intervals (95% CI) were calculated for prevalence comparisons. Variations in biofilm biomass were assessed by one-way analysis of variance (ANOVA). Results were considered statistically significant when *p* values were below 0.05.

## 3. Results

### 3.1. Prevalence of Staphylococcus aureus

From the 105 food handlers’ hands and 156 ready-to-eat food samples, a total of 29 presumptive coagulase-positive staphylococci isolates were recovered. All isolates were subsequently confirmed as *S. aureus* by detection of the *nuc* gene. Of these, 20 were obtained from food handlers’ hands and 9 from RTE foods, corresponding to an overall prevalence of 11.11% (29/261).

The prevalence of *S. aureus* was 19.05% (20/105) in hand swab samples and 5.77% (9/156) in RTE foods. A significantly higher prevalence was observed in food handlers’ hands compared with RTE foods (Fisher’s exact test, *p* = 0.001; OR = 3.84, 95% CI: 1.67–8.82).

Among food handlers, 17 isolates were obtained from university 1 (29.82%; 17/57) and 3 from university 2 (6.25%; 3/48). All *S. aureus* isolates from RTE foods were recovered from university 1.

### 3.2. Virulence Genes

The occurrence of virulence-related genes, including those encoding staphylococcal enterotoxins (*sed*, *seg*, *sei*), toxic shock syndrome toxin (*tst*), and alpha-hemolysin (*hla*), was investigated in 29 *S. aureus* isolates recovered in this study, comprising 20 isolates from hands and 9 from RTE foods. The *nuc* gene was detected in all isolates, confirming their identification as *S. aureus*. The distribution of the virulence genes detected is summarised in Table 2.

The *sei* and *hla* genes were detected in all isolates (100%; 29/29). The *seg* and *tst* genes were detected in 93.10% (27/29) of isolates, while the *sed* gene was not detected in any isolate. Most isolates (86.21%; 25/29) carried four virulence genes (*hla*, *sei*, *seg*, and *tst*), while 13.79% (4/29) harboured three genes. Among isolates with three genes, two genotypes were identified: *hla* + *sei* + *tst* (6.89%; 2/29) and *hla* + *sei* + *seg* (6.89%; 2/29).

Among isolates from food handlers’ hands (*n* = 20), *hla* and *sei* were detected in all isolates (100%; 20/20), followed by *seg* (95%; 19/20) and *tst* (90%; 18/20). The *sed* gene was not detected in any isolate. Regarding isolates from RTE foods (*n* = 9), *hla*, *sei* and *tst* were detected in all isolates (100%; 9/9), while *seg* was detected in 88.89% (8/9). The *sed* gene was not detected in any isolate.

The high prevalence of *sei* and *seg* genes, together with the absence of *sed* among the isolates examined, is consistent with the genetic organisation of the enterotoxin gene cluster (*egc*), in which these genes are frequently co-located and inherited together [8].

Table 3 shows the distribution of CoPS across RTE food categories and the corresponding virulence profiles. The highest prevalence was observed in pâté sandwiches (surimi and tuna) with mayonnaise and fresh parsley (26.1%; 6/23), followed by breaded chicken (14.3%; 1/7), rice (12.5%; 1/8), and burgers (9.1%; 1/11). On food handlers’ hands, CoPS prevalence reached 19.05% (20/105).

Across all isolates, *hla* and *sei* were detected in 100% (29/29), while *seg* and *tst* were detected in 93.10% (27/29). Among food isolates, *tst* was detected in 100% (9/9), whereas *seg* was detected in 88.89% (8/9), while in hand isolates, *seg* and *tst* were detected in 95.00% (19/20) and 90.00% (18/20), respectively. The *sed* gene was not detected in any isolate.

### 3.3. Biofilm-Forming Ability of S. aureus Isolates

A total of 29 *S. aureus* isolates were evaluated for biofilm-forming ability. Overall, 44.83% (13/29) of isolates were classified as biofilm producers, predominantly with weak (24.14%; 7/29) and moderate (20.69%; 6/29) biofilm-forming capacity. No isolate was classified as a strong biofilm producer.

**Figure 1:**
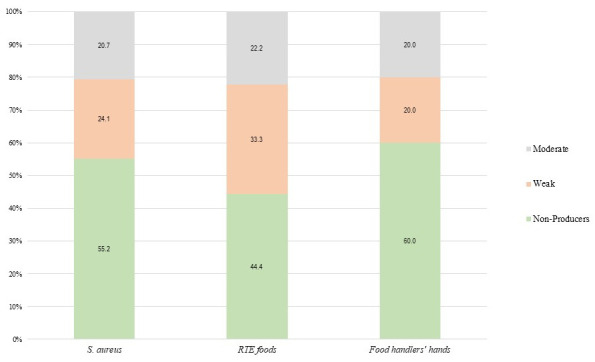
Distribution of non-, weak-, and moderate-biofilm-producing *S. aureus* isolates recovered from RTE foods and food handlers’ hands (%). No strong biofilm producers were detected.

Figure 1 shows the distribution of biofilm-forming capacity among *S. aureus* isolates recovered from ready-to-eat foods and food handlers’ hands.

### 3.4. Biofilm Biomass

**Figure 2:**
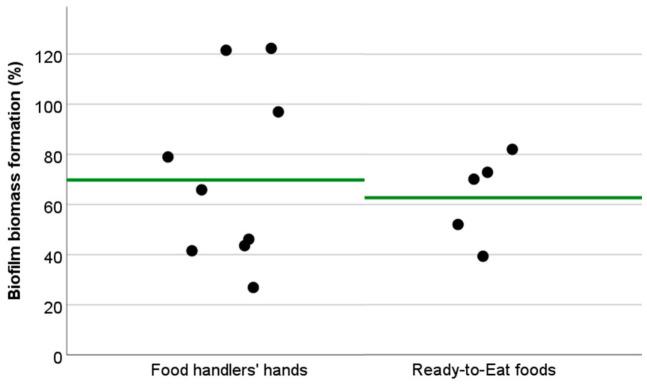
Biofilm biomass of *S. aureus* isolates recovered from RTE foods and food handlers’ hands after 24 h of incubation. The green lines represent the mean biofilm biomass of each group. Values were normalised against the *S. aureus* ATCC^®^ 29213 reference strain.

Mean biofilm biomass, normalised against the *S. aureus* ATCC^®^ 29213 reference strain, was 62.69 ± 14.64 for RTE food isolates and 69.71 ± 36.42 for isolates from food handlers’ hands, with no statistically significant difference observed between the two sources (*p* > 0.05). Figure 2 shows the distribution of individual biofilm biomass values formed after 24 h by *S. aureus* isolates recovered from ready-to-eat foods and food handlers’ hands, with values normalised against the ATCC^®^ 29213 reference strain.

### 3.5. Antimicrobial Resistance

Antimicrobial resistance of *S. aureus* isolates from RTE foods and food handlers’ hands varied across different antibiotic classes. As shown in Table 4, resistance to penicillin G ranged from 11.1% (1/9) in food isolates to 20.0% (4/20) in isolates from food handlers, with an overall prevalence of 17.2% (5/29). No statistically significant differences in antimicrobial resistance frequencies were observed between food and hand isolates (Fisher’s exact test, *p* > 0.05).

Resistance to cefoxitin and imipenem was observed in 13.8% (4/29) of isolates, being more frequent among food handlers’ hands than food samples. Gentamicin resistance was also detected in 17.2% (5/29) of isolates, with a higher prevalence in food samples (33.3%; 3/9). Mupirocin resistance was identified in 13.8% (4/29) of isolates.

Other antibiotic classes, including rifamycins, tetracyclines, macrolides, and lincosamides, showed resistance frequencies ranging between 6.9% (2/29) and 13.8% (4/29) of isolates. No resistance was observed to linezolid, chloramphenicol, or ciprofloxacin (0%; 0/29), indicating retained susceptibility among the isolates.

All resistance phenotypes observed in isolates from both RTE foods and food handlers’ hands were detected only in strains recovered from university 1.

According to the definition proposed by Magiorakos et al. (2012) [37], bacterial isolates are classified as multidrug-resistant (MDR) when exhibiting resistance to at least one agent in three or more antimicrobial classes. Based on this criterion, four isolates (13.79%) were classified as presumptive MRSA and, simultaneously, as MDR (Table 5).

The food isolate SA.F.5, obtained from surimi pâté with mayonnaise and parsley, showed resistance to eight antibiotics across six antimicrobial classes. The remaining three isolates originated from food handlers’ hands. SA.H.13 showed resistance to eight antibiotics across six antimicrobial classes, SA.H.14 to four antibiotics across four antimicrobial classes, and SA.H.15 to nine antibiotics across seven antimicrobial classes.

## 4. Discussion

The detection of *S. aureus* in both RTE foods and food handlers’ hands reinforces the relevance of human handling as a contamination source in university food service environments. Since *S. aureus* is commonly associated with human skin, nasal cavities, and hands, its occurrence in RTE foods is generally interpreted as an indicator of post-processing contamination and hygiene failures during food preparation or service.

In the present study, *S. aureus* was detected in 11.11% (29/261) of samples, including 9 isolates recovered from RTE foods and 20 from food handlers’ hands. Previous microbiological surveillance conducted in the same university food services reported higher contamination levels in cold RTE foods compared with hot meals, with salads showing the highest microbial counts and more frequent detection of hygiene indicators and foodborne pathogens, including *S. aureus* [38]. Those findings were attributed to the absence of a final heat treatment, extensive manual handling, and the potential for cross-contamination during preparation and service.

The current results are consistent with this interpretation and support that handling practice may contribute to the dissemination of *S. aureus* within these food service environments This interpretation is further supported by the environmental study previously conducted in the same universities, where coagulase-positive staphylococci (CoPS) were detected exclusively on food handlers’ hands from university 1, while no CoPS were identified in university 2 [39]. This finding suggests that local hygiene conditions and operational practices may influence contamination dynamics.

Although the microbial loads detected in the previous food quality study in universities food service were generally low [38,40], these findings should be interpreted cautiously, as inadequate temperature control or prolonged storage may allow bacterial growth and subsequent enterotoxin production [41,42]. In the present study, the presence of enterotoxigenic strains remains relevant from a food safety perspective because staphylococcal food poisoning results from the ingestion of preformed enterotoxins rather than viable cells alone. Furthermore, staphylococcal enterotoxins are heat-stable and may persist even after subsequent thermal processing.

The virulence profile observed in the present study demonstrated a high prevalence of toxin-associated genes, particularly *hla*, *seg*, *sei*, and *tst*. These genes were identified in isolates recovered from both foods and handlers indicating the circulation of strains carrying virulence-associated genetic determinants within the university food service environment. PCR detection confirms the presence of these genetic determinants but does not demonstrate gene expression or toxin production.

The absence of the classical *sed* gene contrasts with some food poisoning studies but agrees with reports indicating increasing prevalence of non-classical enterotoxins in food-associated *S. aureus* strains. All isolates carried the *hla* and *sei* genes (100%; 29/29), while *seg* and *tst* were detected in 93.10% (27/29) of isolates; *sed* was not detected. Among isolates recovered from food handlers’ hands, *hla* and *sei* were identified in all strains, followed by *seg* (95%; 19/20) and *tst* (90%; 18/20). In isolates recovered from RTE foods, *hla*, *sei*, and *tst* were detected in all cases (100%; 9/9), whereas *seg* was present in 88.89% (8/9). The high prevalence of *sei* and *seg*, together with the absence of *sed*, is consistent with reports describing the frequent occurrence of *egc*-associated genes in food-related *S. aureus* isolates [8].

Similar variability in enterotoxin gene distribution has been reported in both food handlers and RTE foods. Fernandes et al. [43] reported the presence of *sea* and *tst* in food handler isolates without detection of *sed*, whereas Puah et al. [31] and Mekhloufi et al. [44] identified variable frequencies of *seg* and *sei* among RTE food isolates. In Portugal, Pereira et al. [45] also demonstrated the widespread occurrence of enterotoxin genes in food-associated *S. aureus* isolates.

From a food safety perspective, the primary hazard associated with *S. aureus* is toxin-mediated rather than the mere presence of the microorganism. Regulation (EC) Nº 2073/2005 [46] establishes microbiological criteria for staphylococcal enterotoxins mainly in dairy products. However, staphylococcal food intoxication is frequently associated with ready-to-eat foods and other highly handled products, which are not fully covered by specific criteria. This highlights a limitation in current regulatory contexts, particularly for highly handled foods lacking a final heat treatment step.

Biofilm formation was observed in 44.83% of isolates, although most strains were classified as weak or moderate producers. As biofilm formation was evaluated under in vitro conditions and biofilm biomass was estimated using the crystal violet assay, these findings should be interpreted as an estimate of biofilm-forming capacity rather than a direct measure of persistence under real food service conditions. Similar distributions have been reported in food-associated *S. aureus* isolates [47,48] and are consistent with observations of limited biofilm maturation under standardised laboratory conditions [49]. Nevertheless, even moderate biofilm-forming capacity may contribute to persistence on surfaces and increased tolerance to environmental stresses and sanitation procedures [50]. This characteristic may favour the potential persistence of *S. aureus* in institutional kitchens, particularly on frequently handled surfaces and equipment. From a food safety perspective, biofilms are associated with increased persistence on food-contact surfaces, reduced effectiveness of cleaning and disinfection procedures, and recurrent contamination events [51,52,53].

The occurrence of antimicrobial-resistant *S. aureus* in the food chain represents a potential public health concern due to the possible dissemination of resistance determinants [54]. In the present study, antimicrobial resistance was detected in 31.0% of isolates, including presumptive MRSA and multidrug-resistant strains. Among isolates recovered from food handlers’ hands, resistance was most frequently observed for penicillin G (20%) and mupirocin (15%), whereas isolates from RTE foods showed higher resistance frequencies to gentamicin (33.3%) and tetracycline (22.2%).

Resistance to penicillin G in *S. aureus* is commonly associated with *β*-lactamase production and does not necessarily indicate methicillin resistance. In contrast, resistance to cefoxitin and oxacillin was used as a surrogate marker for methicillin-resistant *Staphylococcus aureus*, associated with *mecA*-mediated resistance. Four isolates (13.79%) were classified as multidrug-resistant, all of which also exhibited resistance to cefoxitin and oxacillin and were therefore classified as presumptive MRSA.

Similar findings have been reported previously. Castro et al. [55] observed high resistance rates to penicillin (64%) and erythromycin (50%) among food-associated *S. aureus* isolates, although no MRSA strains were identified. Saber et al. [56] also reported multidrug-resistant profiles in *S. aureus* isolated from RTE meat products, including resistance to multiple antimicrobial classes. In the present study, the isolate recovered from burger samples (9.1%; 1/11) exhibited resistance to both gentamicin and tetracycline.

The resistance profiles observed among cefoxitin- and oxacillin-resistant isolates are consistent with methicillin resistance mechanisms typically associated with the presence of *mec*A and production of *PBP2a* [57,58], although the *mec*A gene was not directly assessed in this study. Variability in resistance profiles among isolates may reflect differences in genetic determinants and previous antimicrobial exposure [59,60,61]. However, considering the relatively low prevalence of *S. aureus* detected in RTE foods, these products are unlikely to represent a major route for antimicrobial resistance transmission.

The findings suggest that *S. aureus* contamination in these university food services is likely multifactorial, involving direct human handling, environmental persistence, and operational hygiene conditions. These results reinforce the importance of continuous hygiene training, effective hand hygiene practices, environmental monitoring, and adequate sanitation procedures to reduce cross-contamination risks in institutional catering systems.

## 5. Conclusions

This study demonstrated the occurrence of *S. aureus* in both RTE foods and food handlers’ hands, suggesting post-processing contamination pathways in university food service environments. Although microbial loads were low, the detection of virulence-associated genes (*hla*, *sei*, *seg*, and *tst*) indicates the circulation of isolates carrying virulence-associated genes. The findings reinforce the role of food handlers as important contamination sources and emphasise the importance of effective hygiene and handling practices to prevent cross-contamination. Biofilm-forming capacity was predominantly weak to moderate, suggesting a potential contribution to environmental persistence under inadequate sanitation conditions. Antimicrobial resistance, including presumptive MRSA and multidrug-resistant strains, was also identified, although its food safety relevance remains secondary to enterotoxin production. Overall, these results highlight the importance of temperature control, minimising post-processing handling, effective sanitation procedures, and continuous hygiene training to reduce contamination risks in institutional food service environments.

## Figures and Tables

**Table 1:** Sequences of the primers used for the detection of virulence genes in *S. aureus* strains, amplicon sizes, and annealing temperature (*a*).

Name	Primer Sequences	Amplicon Size (bp)	*a* °C	Reference
*nuc*	Forward: 5′GCGATTGATGGTGATACGGTT3′	270	60	[30]
	Reverse: 5′AGCCAAGCCTTGACGAACTAAAGC3′			
*hla*	Forward: 5′CTGATTACTATCCAAGAAATTCGATTG3′	209	59	[31]
	Reverse: 5′CTTTCCAGCCTACTTTTTTATCAGT3′			
*tst*	Forward: 5′ACCCCTGTTCCCTTATCATC3′	326	59	[32]
	Reverse: 5′TTTTCAGTATTTGTAACGCC3′			
*sed*	Forward: 5′GTGGTGAAATAGGACTGC3′	171	59	[33]
	Reverse: 5′ATATGAAGGTGCTCTGTGG3′			
*seg*	Forward: 5′AAGTAGACATTTTTGGCGTTCC3′	287		
	Reverse: 5′AGAACCAGTCCATCTCCTG3′			
*sei*	Forward: 5′CAACTCGAATTTTCAACAGGTACC3′	466		
	Reverse: 5′CAGGCAGTCCATCTCCTG3′			

**Table 2:** Characterization of *S. aureus* isolates and PCR-based detection of virulence genes.

Code Strain	*n*	Source	Virulence Genes
SA.H.01 to 06; SA.H.08; SA.H.10 to 14; SA.H.16 to 20	17	Food handlers’ hands	*hla*, *sei*, *seg*, *tst*
SA.H.07	1		*hla*, *sei*, *tst*
SA.H.09; SA.H.15	2		*hla*, *sei*, *seg*
SA.F.01	1	Rice	*hla*, *sei*, *tst*
SA.F.02 to 07	6	Pâté surimi/tuna sandwich with mayonnaise and fresh parsley	*hla*, *sei*, *seg*, *tst*
SA.F.08	1	Breaded chicken	*hla*, *sei*, *seg*, *tst*
SA.F.09	1	Burger	*hla*, *sei*, *seg*, *tst*

**Table 3:** Prevalence of coagulase-positive staphylococci (CoPS) and distribution of virulence genes in RTE foods and on handlers’ hands in university food service establishments.

Gene	FH Hands (*n* = 20)	RTE Foods			
		Rice (*n* = 1)	Pâté with Mayonnaise and Fresh Parsley (*n* = 6)	Breaded Chicken (*n* = 1)	Burger (*n* = 1)
*hla*	100%	100%	100%	100%	100%
*sed*	0%	0%	0%	0%	0%
*sei*	100%	100%	100%	100%	100%
*seg*	95%	0%	100%	100%	100%
*tst*	90%	100%	100%	100%	100%
CoPS prevalence	20/105 (19.05%)	1/8 (12.5%)	6/23 (26.1%)	1/7 (14.3%)	1/11 (9.1%)

FH: food handlers; RTE: ready-to-eat. Gene frequencies are expressed as percentages among the *S. aureus* isolates recovered from each category (*n*). CoPS prevalence is expressed as positive samples/total samples (%).

**Table 4:** Antimicrobial resistance profiles of *S. aureus* isolates recovered from RTE foods and food handlers’ hands in university food service establishments. Results are expressed as frequency, *n* (%).

Antimicrobial Classes	Antibiotics Tested	Foods	Food Handlers	Total
		*n* (%)	*n* (%)	*n* (%)
Aminoglycosides	Gentamicin	3 (33.3)	2 (10.0)	5 (17.2)
Carbapenems	Imipenem	1 (11.1)	3 (15.0)	4 (13.8)
Cephalosporins	Cefoxitin	1 (11.1)	3 (15.0)	4 (13.8)
Fluoroquinolones	Ciprofloxacin	0 (0.0)	0 (0.0)	0 (0.0)
Lincosamides	Clindamycin	0 (0.0)	2 (10.0)	2 (6.9)
Macrolides	Erythromycin	1 (11.1)	1 (5.0)	2 (6.9)
Oxazolidinones	Linezolid	0 (0.0)	0 (0.0)	0 (0.0)
Penicillins	Oxacillin	1 (11.1)	4 (20.0)	5 (17.2)
	Penicillin G	1 (11.1)	4 (20.0)	5 (17.2)
Phenicols	Chloramphenicol	0 (0.0)	0 (0.0)	0 (0.0)
Pseudomonic acid	Mupirocin	1 (11.1)	3 (15.0)	4 (13.8)
Rifamycins	Rifampicin	1 (11.1)	2 (10.0)	3 (10.3)
Sulfonamides	Sulfamethoxazole/Trimethoprim	0 (0.0)	0 (0.0)	0 (0.0)
Tetracyclines	Tetracycline	2 (22.2)	2 (10.0)	4 (13.8)

Penicillin G (P, 10 U), cefoxitin (FOX, 30 µg), chloramphenicol (C, 30 µg), ciprofloxacin (CIP, 5 µg), clindamycin (DA, 2 µg), erythromycin (E, 15 µg), gentamicin (CN, 10 µg), imipenem (IPM, 10 µg), linezolid (LNZ, 10 µg), mupirocin (MUP, 200 µg), oxacillin (OX, 1 U), rifampicin (RD, 30 µg), tetracycline (TE, 30 µg) and trimethoprim/sulfamethoxazole (SXT, 1.25/23.75 µg).

**Table 5:** Antimicrobial resistance profiles of cefoxitin- and oxacillin-resistant *S. aureus* isolates, all of which were classified as multidrug-resistant (MDR).

Samples	Antibiotic Resistance
SA.F.5	P; OX; FOX; IPM; CN; MUP; RD; E
SA.H.13	P; OX; FOX; IPM; CN; MUP; RD; TE
SA.H.14	OX; FOX; IPM; DA
SA.H.15	P; OX; FOX; IPM; CN; MUP; RD; TE; DA

SA.F.5: food isolate; SA.H.13–15: food handlers’ hand isolates; Penicillin G (P, 10 U), cefoxitin (FOX, 30 µg), clindamycin (DA, 2 µg), erythromycin (E, 15 µg), gentamicin (CN, 10 µg), imipenem (IPM, 10 µg), mupirocin (MUP, 200 µg), oxacillin (OX, 1 U), rifampicin (RD, 30 µg), tetracycline (TE, 30 µg).

## Data Availability

The data presented in this study are available from the corresponding author upon reasonable request. The data are not publicly available due to ethical and confidentiality restrictions related to participant privacy.

## Abbreviations

The following abbreviations are used in this manuscript:

AMR	Antimicrobial resistance
ATCC	American Type Culture Collection
BHI	Brain heart infusion
CoPS	Coagulase-positive staphylococci
DNA	Deoxyribonucleic acid
EFSA	European Food Safety Authority
EU	European Union
ISO	International Organization for Standardization
MDR	Multidrug-resistant
MRSA	Methicillin-resistant *Staphylococcus aureus*
PCR	Polymerase chain reaction
RTE	Ready-to-eat
SA.F	*S. aureus* isolate from a food sample
SA.H	*S. aureus* isolate from a food handler’s hand sample
SE	Staphylococcal enterotoxin
SEl	Staphylococcal enterotoxin-like
SFP	Staphylococcal food poisoning
TSS	Toxic shock syndrome
